# Duration of Social Isolation Affects Production of Nitric Oxide in the Rat Brain

**DOI:** 10.3390/ijms221910340

**Published:** 2021-09-25

**Authors:** Stanislava Vrankova, Zuzana Galandakova, Jakub Benko, Martina Cebova, Igor Riecansky, Olga Pechanova

**Affiliations:** 1 Centre of Experimental Medicine, Institute of Normal and Pathological Physiology, Slovak Academy of Sciences, 841 04 Bratislava, Slovakia; galandakova.zuzana@gmail.com (Z.G.); jakub.benko@savba.sk (J.B.); martina.cebova@savba.sk (M.C.); igor.riecansky@savba.sk (I.R.); olga.pechanova@savba.sk (O.P.); 2 Social, Cognitive and Affective Neuroscience Unit, Department of Cognition, Emotion, and Methods in Psychology, Faculty of Psychology, University of Vienna, 1010 Vienna, Austria; 3 Department of Psychiatry, Slovak Medical University, 833 03 Bratislava, Slovakia

**Keywords:** social isolation, neurodevelopment, nitric oxide, nitric oxide synthase, oxidative stress

## Abstract

Social isolation deprives rodents of social interactions that are critical for normal development of brain and behavior. Several studies have indicated that postweaning isolation rearing may affect nitric oxide (NO) production. The aim of this study was to compare selected behavioral and biochemical changes related to NO production in the brain of rats reared in social isolation for different duration. At the age of 21 days, male Sprague Dawley rats were randomly assigned into four groups reared in isolation or socially for 10 or 29 weeks. At the end of the rearing, open-field and prepulse inhibition (PPI) tests were carried out. Furthermore, in several brain areas we assessed NO synthase (NOS) activity, protein expression of nNOS and iNOS isoforms and the concentration of conjugated dienes (CD), a marker of oxidative damage and lipid peroxidation. Social isolation for 10 weeks resulted in a significant decrease in PPI, which was accompanied by a decrease in NOS activity in the cerebral cortex and the cerebellum, an increase in iNOS in the hippocampus and an increase in CD concentration in cortex homogenate. On the other hand, a 29 week isolation had an opposite effect on NOS activity, which increased in the cerebral cortex and the cerebellum in animals reared in social isolation, accompanied by a decrease in CD concentration. The decrease in NOS activity after 10 weeks of isolation might have been caused by chronic stress induced by social isolation, which has been documented in previous studies. The increased oxidative state might result in the depleted NO bioavailability, as NO reacts with superoxide radical creating peroxynitrite. After 29 weeks of isolation, this loss of NO might be compensated by the subsequent increase in NOS activity.

## 1. Introduction

The chronic psychosocial stress during the early post-natal phases plays an important role in brain development. Numerous data have reported that exposure to chronic early-life stress is associated with development of neuroinflammatory disorders later in adulthood, such as neurological and psychiatric diseases. Previous studies have demonstrated that social isolation stress could induce depressive- and anxiety-like behaviors, learning deficits and memory impairments through affecting cell proliferation, neurogenesis, mitochondrial dysfunction and alteration in release of neurotransmitters in the hippocampus and prefrontal cortex [1,2,3]. It is of great importance to understand the mechanisms underlying psychopathological states induced by adverse early-life experiences. Converging lines of evidence suggest that post-weaning isolation rearing of rodents is an animal model that mimics some of the behavioral consequences of stressful early-life experiences in humans. Animals reared in social isolation exhibit many abnormalities in behavior and altered brain neurochemistry [4,5]. Most of the research has been conducted on rodents and is performed by simple physical isolation of individuals from their home cages. Generally, animals are separated either for a short time (e.g., several hours or overnight), or longer periods of continual separation (e.g., days or weeks). As such, it induces a robust stress response comprising of systemic changes in catecholamine, corticosterone, adrenocorticotropin, corticotropin-releasing factor concentration, autonomic hyperarousal, and changes in cytokine profile [6,7]. In the brain, it leads to changes in monoamine, glutamate, GABA and steroid neurotransmission, and expression of brain-derived neurotrophic factor (BDNF) [8,9,10]. At a behavioral level, it provokes aggression, depression-like phenotype or various endophenotypes of schizophrenia [7,11,12].

Nitric oxide (NO) is an important signalling molecule in the nervous system and participates in a host of functions and processes including regulation of vascular tone, immune response, and synaptic connectivity. In the brain, NO is involved in neuronal migration, formation of synapses and regulation of neurotransmission [13,14,15]. Neuronal nitric oxide synthase (nNOS) is a principal isoform expressed in the brain and accounts for more than 90% of NO production [16]. At glutamatergic synapses, post-synaptic neuronal activation with subsequent activation of N-methyl-D-aspartate (NMDA) receptors leads to calcium influx. This in turn leads to activation of nNOS in a Ca^2+^/calmodulin dependent manner. Released NO exerts its effect pre- and post-synaptically via activation of soluble guanylate cyclase [17]. Among many regions, prefrontal cortex, basal ganglia, limbic structures, and monoaminergic nuclei, were found to be expressing nNOS. Changes in NO production and/or nNOS expression have been implicated in the etiopathology of various neurological or psychiatric diseases, including Parkinson’s disease, Alzheimer’s disease, major depression, and schizophrenia [18,19,20].

NO may have beneficial or detrimental effects, primarily depending on the oxidation state in the cell. The physiological production of NO acts neuroprotectively and ensures proper operation of nerve functions. On the other hand, NO overload may trigger its operation as a free radical and contribute to cell membrane damage [21].

Chronic systemic immune activation might trigger neuroinflammation in the brain including upregulation of inducible NOS (iNOS) in immune-system-derived neuroglia [22]. Thus, NO produced by these cells might leak to neurons and interfere with normal regulation of neuroplasticity. Previously, nitrergic system abnormalities, increased oxidative state and neurodegeneration have been implicated in behavioral changes accompanying social isolation [12,23].

The purpose of this study was to investigate the effect of social isolation rearing on NO production in various rat brain areas as well as oxidative damage. Furthermore, we explored how these alterations related to behavioral changes after 10 week and 29 week periods of social isolation.

## 2. Results

### 2.1. Behavioral Tests

**Figure 1:**
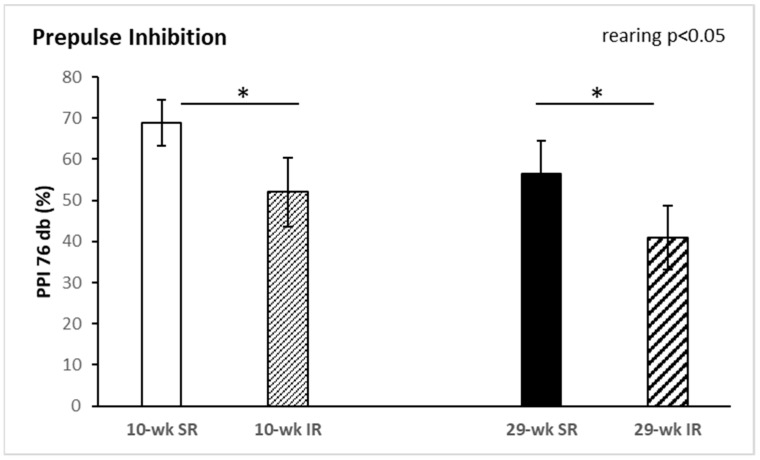
Effects of isolation rearing conditions on pre-pulse inhibition at 76 dB level. 10–wk SR, 10 week socially reared SD rats; 10–wk IR, 10 week isolation reared SD rats; 29–wk SR, 29 week socially reared SD rats; 29–wk IR, 29 week isolation reared SD rats; SD, Sprague Dawley; PPI, pre-pulse inhibition. Data are expressed as the mean ± SEM. Statistical significance as revealed by two-way ANOVA with subsequent Sidak post-hoc test when appropriate: * *p* < 0.05.

Rearing conditions or duration had no significant effect on the behavior in the open-field test (data not shown). For PPI, a two-way ANOVA revealed a significant main effect of rearing at 76 dB pre-pulse intensity (F_(1, 34)_ = 4.609, *p* < 0.05, η^2^ = 0.119). As shown in Figure 1, PPI was significantly decreased in animals reared in social isolation. The interaction between duration and rearing was not significant. Startle reactivity and startle habituation were not significantly affected by rearing conditions or duration (data not shown, see Supplementary Materials Tables S1–S5 for more details).

### 2.2. Total Activity of NOS

**Figure 2:**
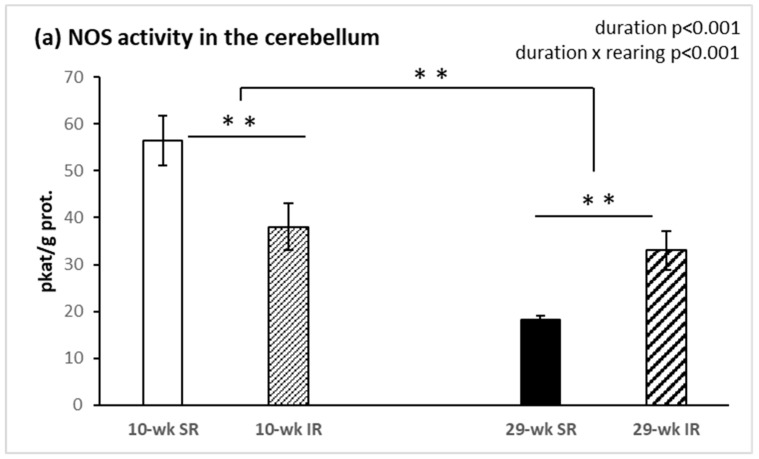

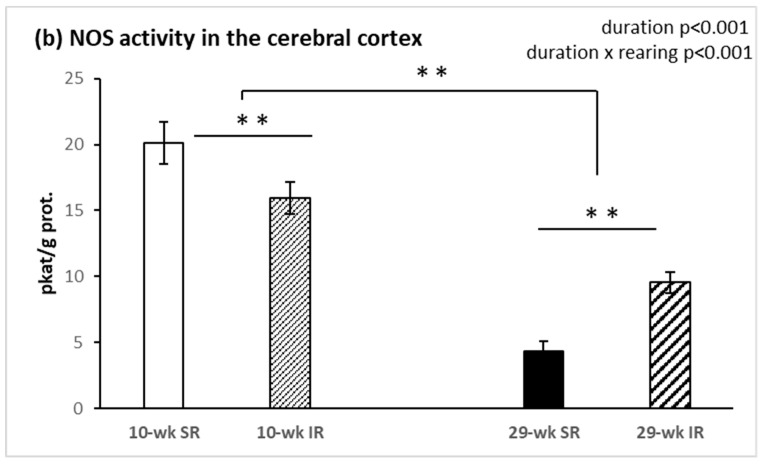
Effects of isolation rearing conditions on NOS activity in the cerebellum (**a**), and frontal cortex posterior to PFC (**b**). 10–wk SR, 10 week socially reared SD rats; 10–wk IR, 10 week isolation reared SD rats; 29–wk SR, 29 week socially reared SD rats; 29–wk IR, 29 week isolation reared SD rats; NOS, nitric oxide synthase; PFC, prefrontal cortex; SD, Sprague Dawley. Data are expressed as the mean ± SEM. Statistical significance as revealed by two-way ANOVA with subsequent Sidak post-hoc test when appropriate: ** *p* < 0.001.

The total NOS activity in the cerebellum was significantly affected by the duration of rearing (F_(1, 34)_ = 31.85, *p* < 0.001, η^2^ = 0.484), being overall lower after 29 weeks compared with 10 weeks of rearing. Notably, however, the effect of social isolation depended on rearing duration, as indicated by a significant condition × duration interaction (F_(1, 34)_ = 18.83, *p* < 0.001, η^2^ = 0.356). When compared with socially housed animals, isolated rats showed significantly decreased cerebellar NOS activity after 10 weeks, but increased activity after 29 weeks of rearing (both *p* < 0.05, Figure 2a). Similar effects on NOS activity were found in the frontal cortex (Figure 2b), including a significant main effect of rearing duration (F_(1, 34)_ = 101.54, *p* < 0.001, η^2^ = 0.749) and a significant interaction between rearing conditions and duration (F_(1, 34)_ = 18.18, *p* < 0.001, η^2^ = 0.348).

### 2.3. Protein Expression of nNOS and iNOS

**Figure 3:**
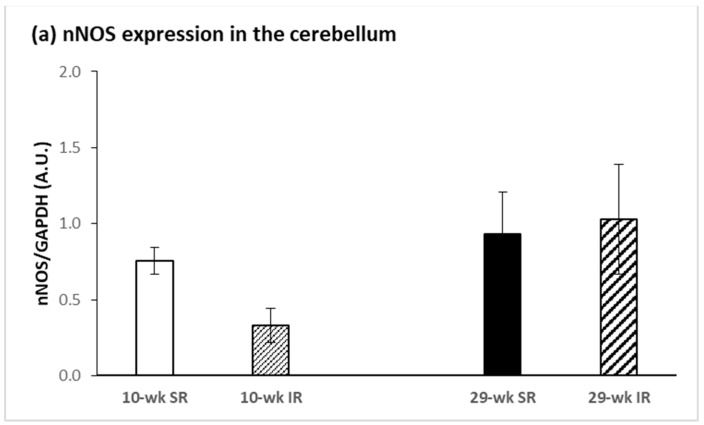

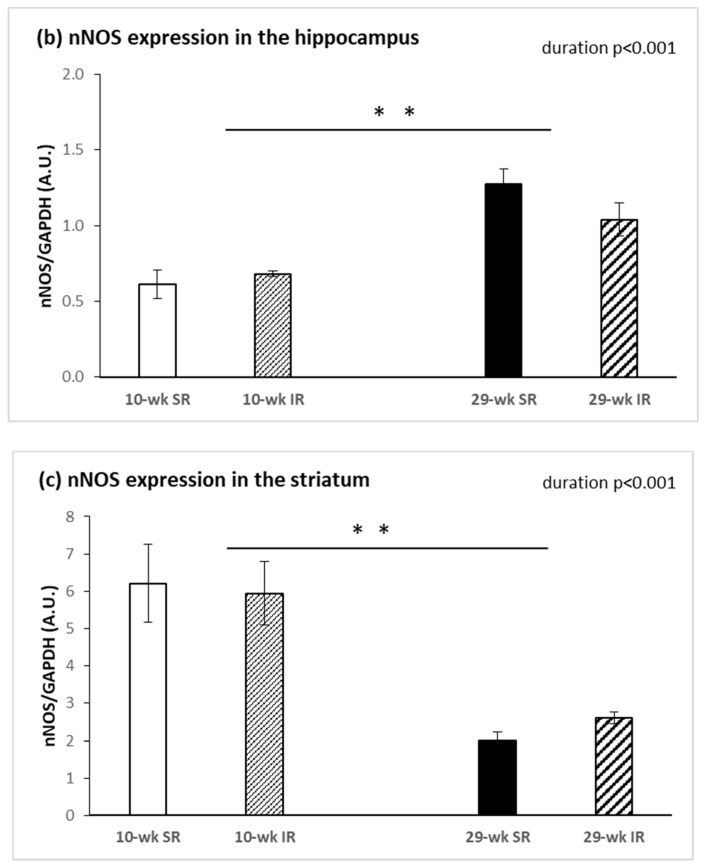
Effects of isolation rearing conditions on protein expression of nNOS in the cerebellum (**a**), hippocampus (**b**), and striatum (**c**). 10–wk SR, 10 week socially reared SD rats; 10–wk IR, 10 week isolation reared SD rats; 29–wk SR, 29 week socially reared SD rats; 29–wk IR, 29 week isolation reared SD rats; nNOS, neuronal nitric oxide synthase; iNOS, inducible nitric oxide synthase; SD, Sprague Dawley; GAPDH, glyceraldehyde 3–phosphate dehydrogenase; A.U., arbitrary units. Data are expressed as the mean ± SEM. Statistical significance as revealed by two-way ANOVA with subsequent Sidak post-hoc test when appropriate: ** *p* < 0.001.

Social isolation had no significant effect on protein expression of nNOS in the cerebellum, hippocampus, striatum, or frontal cortex. Rearing duration had a significant effect only in the hippocampus and the striatum, but the effects were in the opposite direction. In the hippocampus, nNOS expression was significantly increased after 29 weeks compared with 10 weeks of rearing (F_(1, 28)_ = 24.051, *p* < 0.001, η^2^ = 0.462) (Figure 3b). In the striatum, in contrast, 29 weeks of rearing resulted in decreased nNOS expression when compared with 10 weeks of rearing (F_(1, 28)_ = 48.564, *p* < 0.001, η^2^ = 0.634) (Figure 3c).

**Figure 4:**
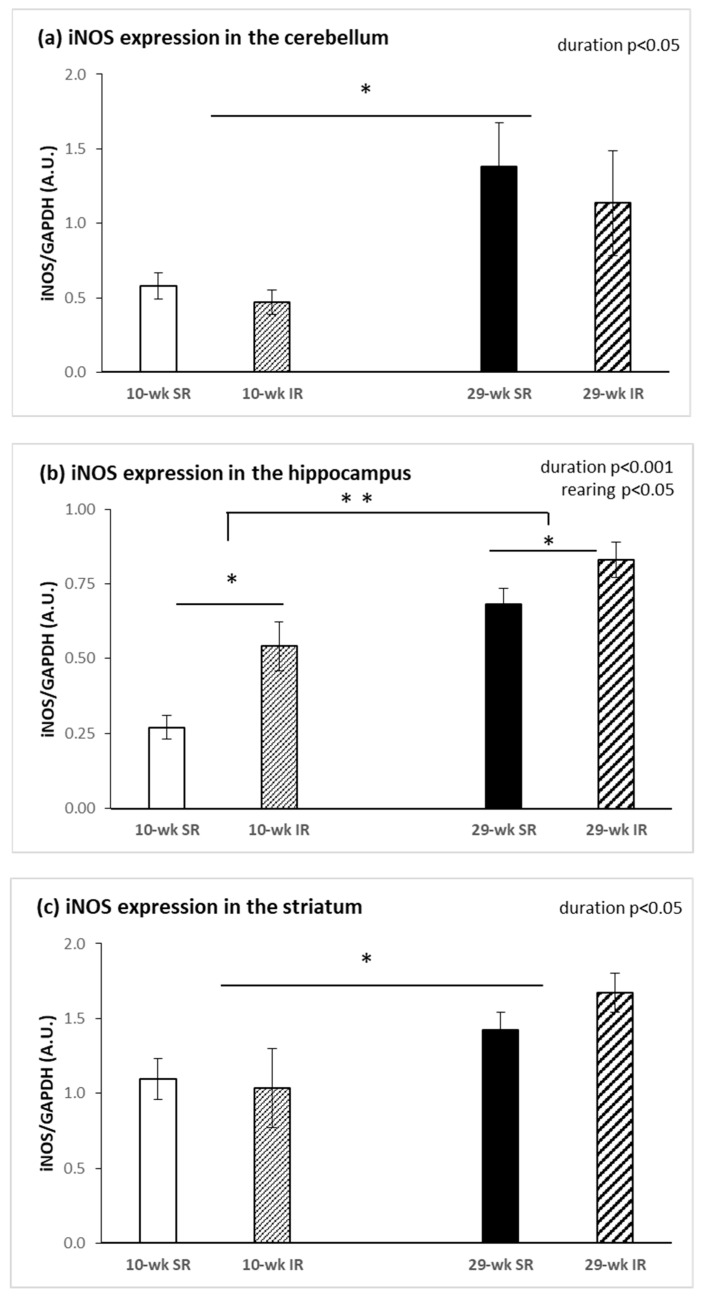
Effects of isolation rearing conditions on protein expression of iNOS in the cerebellum (**a**), hippocampus (**b**) and striatum (**c**). 10–wk SR, 10 week socially reared SD rats; 10–wk IR, 10 week isolation reared SD rats; 29–wk SR, 29 week socially reared SD rats; 29–wk IR, 29 week isolation reared SD rats; nNOS, neuronal nitric oxide synthase; iNOS, inducible nitric oxide synthase; SD, Sprague Dawley; GAPDH, glyceraldehyde 3–phosphate dehydrogenase; A.U., arbitrary units. Data are expressed as the mean ± SEM. Statistical significance as revealed by two-way ANOVA with subsequent Sidak post-hoc test when appropriate: * *p* < 0.05, ** *p* < 0.001.

Rearing rats for 29 compared with 10 weeks significantly increased protein expression of iNOS in the cerebellum (F_(1, 28)_ = 5.754, *p* < 0.05, η^2^ = 0.170), hippocampus (F_(1, 28)_ = 30.386, *p* < 0.001, η^2^ = 0.520), and striatum (F_(1, 28)_ = 8.898, *p* < 0.05, η^2^ = 0.229). A significant effect of isolation was only revealed in the hippocampus (F_(1, 28)_ = 11.081, *p* < 0.05, η^2^ = 0.284): the expression was higher in isolated, compared with socially, reared animals (Figure 4b). No significant effects on iNOS protein expression were revealed in the frontal cortex.

### 2.4. Conjugated Dienes Concentration

**Figure 5:**
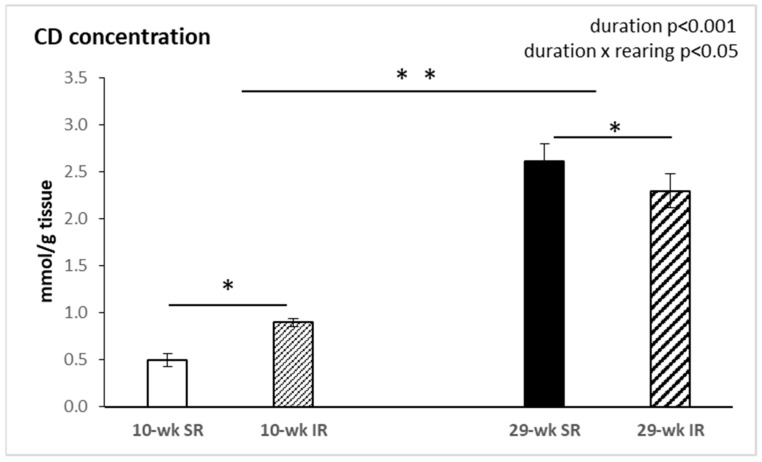
Effects of isolation rearing conditions on concentration of conjugated dienes in cortex posterior to PFC. 10–wk SR, 10 week socially reared SD rats; 10–wk IR, 10 week isolation reared SD rats; 29–wk SR, 29 week socially reared SD rats; 29–wk IR, 29 week isolation reared SD rats; PFC, prefrontal cortex; SD, Sprague Dawley. Data are expressed as the mean ± SEM. Statistical significance as revealed by two-way ANOVA with subsequent Sidak post-hoc test when appropriate: * *p* < 0.05, ** *p* < 0.001.

As shown in Figure 5, concentration of CDs in the frontal cortex was significantly affected by duration of rearing (F_(1, 34)_ = 145.88, *p* < 0.001, η^2^ = 0.807), showing significantly higher levels in animals reared for 29 weeks. There was also a significant interaction between rearing conditions and duration (F_(1, 34)_ = 6.07, *p* < 0.05, η^2^ = 0.148).

## 3. Discussion

The most important finding of this study is that the activity of nitric oxide synthase in the brain has been affected by the duration of social isolation. Ten week social isolation decreased NOS activity, while a two-fold increase in NOS activity was observed in 29 week isolated animals. Conjugated dienes concentration, a marker of lipid peroxidation, was raised after 10 weeks, and decreased after 29 weeks, of isolation rearing.

From a disease standpoint, multiple preclinical and clinical findings have linked the disturbed NO signaling to pathophysiology of psychiatric disorders characterized by sensorimotor gating deficits [18,24,25]. Our results from sensorimotor gating assessment showed significantly decreased PPI in animals reared in social isolation, as has been shown previously [26]. Similarly, no differences in startle magnitude were observed. Consistent with our previous study, isolation reared animals exhibited significant deficits in PPI compared with socially reared animals [27]. The results of the present study demonstrate that NOS activity in the cerebellum and cerebral cortex was significantly affected by duration of social isolation with significantly lower NOS activity after 29 weeks. We have seen also a swing in NOS activity in cerebellum and frontal cortex between 10 week and 29 week isolation rearing. More specifically, we observed a significantly lower NOS activity in 10 week IR rats and a higher NOS activity in 29 week IR rats. There is a growing body of evidence that supports the role of NO in PPI regulation [28,29,30]. It is well documented that the NMDA receptor strongly interacts with the NO/cGMP system. Activation of postsynaptic neuronal NMDA receptors in several brain areas elevates the levels of intracellular Ca^2+^ ions, which triggers the activity of Ca-calmodulin followed by nNOS stimulation and subsequent increases of NO levels. Moreover, it was reported that PSD-95 protein accelerates the interaction of the NMDA receptor and NOS through Ca^2+^ influx, and hence augments the NO production [31,32]. NOS inhibitors have been observed to attenuate PPI deficits mediated by dopamine receptor activation [33] or NMDARs antagonism [28,29]. The link between dysregulated dopaminergic function and sensorimotor gating abnormalities in psychiatric diseases [34,35,36] has been well considered and consolidated over past decades. Previous investigations have reported a more important role for D1 receptors than D2 receptors in PPI regulation [37]. Moreover, an activation of D1 receptors alone might not be sufficient to disrupt PPI function in rats, but the ability of D2 receptors to induce PPI dysfunction is dependent upon the tonic activity of D1 receptors. Hence, based on these findings, it appears that D1 receptors might be interacting with D2 receptors in the modulation of PPI alterations [36]. Overall, based on findings from previous reports, the regulation of NOS activity can contribute to dopaminergic control of PPI deficits [38]. We hypothesized that increased NO levels after 29 week IR could be involved in D1 receptor stimulation as well as in PPI regulation. It is possible that reciprocal D1-NMDA receptor interactions play a critical role in regulating striatal nNOS interneuron activity.

It has been shown that genetic deficiency of nNOS generates a mouse model that exhibits behavioral and neurochemical abnormalities that are reminiscent of psychiatric disorders [39]. Our findings revealed a tendency toward lower nNOS protein levels in 10 week isolation reared animals, but the difference did not reach statistical significance. Duration specific patterns of nNOS protein expression revealed increased nNOS in the hippocampus and decreased nNOS in the striatum in 29 week groups compared with 10 week groups. The results of our previous study demonstrated attenuated mRNA levels of nNOS, VGF and TrkB receptor in the hippocampus after post-weaning social isolation [27]. In the present study, 10 week post-weaning social isolation had no significant effect on the protein expression of nNOS in the hippocampus, but it induced an increase in hippocampal iNOS protein levels. Zlatkovic et al. also showed increased iNOS expression in the hippocampus and the prefrontal cortex of male rats exposed to the chronic stress of isolation [40,41]. Increased iNOS level is considered to be an indicator of stress-induced impairment in the brain [42]. In our study, iNOS protein expression was significantly affected by duration of rearing and it was increased in the cerebellum, in the hippocampus and also in the striatum. A previous study reported that exposure to 21 days of chronic social isolation triggers proapoptotic signaling [43] and leads to programmed cell death in the rat prefrontal cortex but not in the hippocampus [44], indicating the existence of some form of protection in the hippocampus. Given that the hippocampus is highly sensitive to stress, Zlatkovic et al. showed signaling cascades underlying the hippocampal cellular protection through the NOS pathway, antioxidant capacity and heat shock protein expression [41]. An increased level of Hsp70i protein may be involved in the inhibition of hippocampal apoptosis in conditions of social isolation. Other studies have indicated that Hsp70i induction protects neurons from apoptosis through its ability to increase Bcl-2 stability during oxidative stress [45] and suppress mitochondrial cytochrome c release [46] or activity of c-Jun N-terminal kinase [47]. In this study, when comparing the duration of social isolation, we detected significantly decreased protein levels for nNOS in the striatum. Since we also detected decreased NOS activity in the cerebellum and frontal cortex, we assumed that decreased nNOS expression was connected with decreased NOS activity. The diffusible nature of NO and the physiological link with NMDA receptor activity in cerebellar circuits contributed to this early identification as an appealing candidate for mechanisms of synaptic plasticity. Since then, evidence for the NO/cGMP involvement in plastic changes of synaptic strength, and thus in processes of learning and remembering, has accumulated to a probably unexpected extent [48]. NO produced by parallel fibers, with a possible contribution of a molecular layer of nNOS-positive neurons, diffuses into Purkinje neurons where it stimulates guanylyl cyclase and activates PKG, contributing to hyperphosphorylation of AMPA receptors which results in their declustering and endocytotic recycling, thus lowering excitatory response to glutamate [49]. The tetrameric AMPA receptor complexes are composed of four subunits, GluR1–4. The GluR4 subunit is highly expressed in the cerebellum and the early postnatal hippocampus and is thought to be involved in synaptic plasticity and the development of functional neural circuitry [50]. Wang and Zhu demonstrated that NO was essential for the survival of differentiating cerebellar granule neurons in vitro, since the blockade of NO production resulted in extensive neuronal death while a NO donor was able to significantly revert this effect. They found that endogenous NO contributed to the survival of differentiating cerebellar granule neurons by a cGMP-dependent mechanism, while the exogenous NO has been proposed to mediate this effect via cGMP-independent mechanisms [51]. Sammut et al. used NO selective microsensors that showed that striatal NO efflux was robustly increased in vivo by electrical stimulation of corticostriatal afferents via an NMDA receptor-dependent mechanism and an nNOS-dependent mechanism [52]. Intrastriatal infusion of NMDA has also been shown to activate NO efflux in vivo [53], indicating that NMDA receptors play a primary role in stimulating nNOS activity. It is also known that dopamine D1 receptor activation stimulated striatal NO synthesis, whereas D2 receptor activation produced the opposite effect [54].

It has been shown that exposure to repeated stress situations increases reactive oxygen species generation in the brain, where NO and an excess of pro-oxidants are responsible for both neuronal functional impairment and structural damage [55]. Redox dysregulation may constitute a background where genetic and environmental factors converge and their timing during neurodevelopment could play a decisive role. The brain is particularly vulnerable to oxidative damage because of its high oxygen utilization, its high content of oxidizable polyunsaturated fatty acids and the presence of redox-active metals. The brain is one of the most lipid-rich organs and the correct lipid turnover or metabolism is important for its proper functioning. Evidence suggests that lipids play a vital role in the composition of the brain and influences its neuronal functioning, including behavior [56]. Free radicals trigger lipid peroxidation of cell membranes and subsequently produce reactive intermediates that can undergo further reactions. This deteriorates the membrane fluidity by increasing its rigidity, leading to impaired receptor function [57]. For instance, dopamine catabolism is distorted by elevated levels of malondialdehyde (MDA). Monoamine oxidase converts dopamine into a corresponding aldehyde. The aldehyde, which is toxic, can accumulate if increased lipid peroxidation produces elevated levels of MDA, forming neurotoxic products [58]. In the present study, 10 week social isolation was observed to significantly increase CD concentration in the frontal cortex of rat brains compared to the control group. Previous studies also observed increased concentrations of MDA and TBARS in plasma, serum, and red blood cells as evidence of membrane oxidative damage [59,60]. Moller et al. reported increased lipid peroxidation, increased SOD activity, and decreased reduced glutathione (GSH) in the brain of animals reared in social isolation. Isolation rearing induced a decrease in activity of cytosolic/nuclear CuZnSOD and mitochondrial MnSOD, as well as GSH levels in the prefrontal cortex, indicating a state of oxidative stress [61]. Moreover, under conditions of intracellular ROS overproduction, cofactors needed for NO synthesis, especially tetrahydrobiopterin, may be oxidized, which leads to uncoupling of the NOS dimer resulting in decreased NOS activity [62]. We also detected decreased NOS activity after 10 week IR. On the other hand, our results showed decreased levels of CD in the frontal cortex after 29 weeks of isolation rearing, whereas NOS activity was significantly increased. These findings indicate that long-term isolation rearing triggers compensation mechanisms, which can effectively improve the oxidative status in the brain and increase NOS activity.

## 4. Materials and Methods

### 4.1. Animals

Adult timed-pregnant *Sprague Dawley* rats (Velaz, Prague, Czech Republic) arrived at the animal facility on gestational day 16. Approximately a week later, the litter was born. Rats were kept under standard housing conditions with a constant 12:12 h light/dark cycle, temperature (22 °C ± 2 °C) and humidity (55 ± 10%). Food and water were available ad libitum. All experimental procedures were performed in accordance with the guidelines of the Institute of Normal and Pathological Physiology, Centre Experimental Medicine Slovak Academy of Sciences (INPP CEM SAS), and were approved by the State Veterinary and Food Administration of the Slovak Republic (Ro-591/17-221) and by an ethics committee according to the European Convention for the Protection of Vertebrate Animals used for Experimental and Other Scientific Purposes, Directive 2010/63/EU of the European Parliament.

### 4.2. Social Isolation

The sample involved 36 male rats. The animals were separated from their mothers after weaning (21 days postnatal) and were randomly divided into four groups. Two experimental groups were subjected to either 10 week or 29 week isolation (single rat per cage, 43.5 × 28 × 23 cm); in two control groups, rats were reared socially for 10 or 29 weeks (three rats per cage, 55.5 × 34.5 × 19.5 cm). In each group, the rats were able to see, smell and hear other animals in the room.

### 4.3. Behavioral Testing

The behavioral tests were carried out at the end of rearing periods. All tests were conducted during the light period of the light/dark cycle. Animals were transported in their home cages from the animal room to the testing room and left undisturbed for 1 h before testing. The experimental sessions were recorded by a video camera positioned above the testing apparatus and behavior was analyzed by a trained observer using a computer program for registration of behavior. At the end of the test procedure, each animal was returned to its home cage.

#### 4.3.1. Open-Field Test

To assess general locomotor activity and anxiety-like behavior, the open-field test was conducted [63]. The animals were tested in a 100 × 100 cm square-shaped black arena with 50 cm high black-painted plywood walls and a black rubber floor, illuminated by a dim light. Each animal was placed in the corner of the open field arena and allowed to freely explore the environment for 10 min. The arena was thoroughly cleaned with 70% ethanol after each animal. Locomotor activity (velocity and distance moved) and anxiety-like behavior (time spent on the peripheral vs. central zone) were evaluated with the program Anymaze (Stoelting Europe, Dublin, Ireland).

#### 4.3.2. Prepulse Inhibition of Startle (PPI) Paradigm

Twenty-four hours following the open-field test, an assessment of changes in sensorimotor gating was performed using the acoustic startle/PPI paradigm [64]. Briefly, the test was performed in a single test station—a sound-attenuating chamber (interior 50.8 × 33 × 30.5 cm; walls 1.9 cm thick) with a plexiglass cylinder situated on the top of a platform including an accelerometric sensor that detected changes in force made by the movements of the rat. Vibrations created by rat body movements were transduced and converted into a signal proportional to response amplitude. Auditory stimuli were delivered by two speakers situated on the sides of the cylinder (Med Associates, Sawbridgeworth, Hertfordshire, UK). The apparatus was thoroughly cleaned with 70% ethanol after each animal. The experimental session consisted of a 5 min acclimatization period to a 65 dB background noise (presented throughout the whole session), followed by three blocks of acoustic trials structured as follows: The first and third block tested for acoustic startle response only, and included 10 pulse-alone trials (120 dB, 38 ms in duration). The second block assessed PPI and contained 28 stimuli, of which 4 were null trials, 4 pulse-alone trials, 4 prepulse-alone and 16 prepulse + pulse trials presented in a pseudo-randomized order. Prepulse stimuli were 18 ms long and presented evenly in intensities of 67, 70, 73 or 76 dB. In the prepulse + pulse trials the pulse was administered 100 ms after prepulse stimulus. Inter-trial interval (ITI) was random in duration with an average of 15 s. The following measures were calculated: Startle reactivity was defined as the mean startle response to pulse-alone trials in first block. Startle habituation (% habituation) was calculated using the formula 100 × (1 − [mean startle for block3/mean startle for block1]). The efficiency of PPI (% PPI) was determined according to the formula 100 × (1 − [startle magnitude on prepulse-pulse trials in block2/startle magnitude on pulse trials in block2]) so that 0% value indicated no difference between the responses to prepulse-pulse trials and pulse alone trials (i.e., no PPI). %PPI was calculated separately for each prepulse intensity along with average %PPI across all prepulse intensities.

### 4.4. Nitric Oxide Synthase Activity Assay

Total NO synthase activity was determined in crude homogenates of the frontal cortex and cerebellum by measuring (3H)-L-citrulline formation from (3H)-L-arginine (ARC, St. Louis, MT, USA) as described elsewhere [65,66]. (3H)-L-citrulline was measured with the Quanta Smart triCarb Liquid Scintillation Analyzer (Packard Instrument Company, Meriden, CT, USA). NOS activity was expressed as pkat/min per gram of protein.

### 4.5. Western Blot Analysis

For Western blot analysis, samples of the tissues (hippocampus, striatum, cerebellum, and frontal cortex) were homogenized in a lysis buffer, 0.05 mM Tris containing protease inhibitor cocktail (Sigma-Aldrich, Taufkirchen, Germany). Protein concentrations were determined by Lowry assay. Proteins were subjected to 10% SDS-PAGE and transferred onto a nitrocellulose membrane. Membranes were blocked with 5% non-fat milk in Tris-buffer solution (TBS; pH 7.6) containing 0.1% Tween-20 (TBS-T) for 1 h at room temperature and then incubated in the presence of the appropriate primary antibodies overnight at 4 °C with polyclonal rabbit anti-neuronal NOS, anti-inducible NOS and anti-GAPDH (as control) antibodies (Abcam, Cambridge, UK). Antibodies were detected using a secondary peroxidase-conjugated antirabbit antibody (Abcam, Cambridge, UK). The bands were visualized using the enhanced chemiluminescence ECL system (Sigma-Aldrich, Taufkirchen, Germany), quantified by using ChemiDoc™ Touch Imagine System (Image Lab™ Touch software, Bio-Rad, Hercules, CA, USA), and normalized to GAPDH bands.

### 4.6. Concentration of Conjugated Dienes

The concentration of conjugated dienes was measured in lipid extracts of the frontal cortex. Samples were homogenized in 15 mmol/dm^3^ EDTA containing 4% NaCl. Lipids were extracted using a 1:1 chloroform-methanol mixture. Chloroform was evaporated in the N2 atmosphere and after the addition of cyclohexane, conjugated diene concentrations were determined spectrophotometrically (λ = 233 nm, NanoDrop^TM^ 2000c, UV-Vis spectrophotometer). The concentration of CD was expressed as nmol per g tissue.

### 4.7. Statistical Analysis

Data were processed and analyzed using JASP 0.14.1.0. All data were analyzed by two-way analysis of variance (ANOVA) with factors rearing condition (social vs. isolation) and rearing duration (10 week vs. 29 week) followed by Sidak post-hoc test when appropriate. Partial eta-squared (η^2^) was used as a measure of effect size. The level of statistical significance was set as *p* < 0.05.

## 5. Conclusions

The present study demonstrates that the activity of nitric oxide synthase in the rat brain depends on the duration of social isolation. Overall, the current investigation provides novel insights into a potential interaction between neuronal NO synthase and oxidative stress in long-term post-weaning social isolation in rats. Our findings indicate that 10 week post-weaning social isolation alters signaling via NO/ROS that could interfere with neurodevelopmental processes which may contribute to pathological behavioral symptoms. On the other hand, this loss of NO after 10 weeks of social isolation might be compensated by the subsequent increase in NOS activity and decrease in ROS after 29 weeks of social isolation. These findings have implications for understanding mechanisms involved in early life stress-related diseases, and demonstrate some improvement in adulthood as a result of compensation mechanisms.

## Data Availability

All data arising from this study are contained within the article and any additional data sharing will be considered by the first author upon request.

## Supplementary Materials

The following are available online at https://www.mdpi.com/article/10.3390/ijms221910340/s1.

## References

[1] Veenema A.H., Reber S.O., Selch S., Obermeier F., Neumann I.D. (2008). Early Life Stress Enhances the Vulnerability to Chronic Psychosocial Stress and Experimental Colitis in Adult Mice. Endocrinology.

[2] Williams A., Umemori H. (2014). The best-laid plans go oft awry: Synaptogenic growth factor signaling in neuropsychiatric disease. Front. Synaptic Neurosci..

[3] Mumtaz F., Khan M.I., Zubair M., Dehpour A.R. (2018). Neurobiology and consequences of social isolation stress in animal model—A comprehensive review. Biomed. Pharmacother..

[4] Schubert M., Porkess M., Dashdorj N., Fone K., Auer D. (2009). Effects of social isolation rearing on the limbic brain: A combined behavioral and magnetic resonance imaging volumetry study in rats. Neuroscience.

[5] Cirulli F., Berry A., Bonsignore L.T., Capone F., D’Andrea I., Aloe L., Branchi I., Alleva E. (2010). Early life influences on emotional reactivity: Evidence that social enrichment has greater effects than handling on anxiety-like behaviors, neuroendocrine responses to stress and central BDNF levels. Neurosci. Biobehav. Rev..

[6] Malkesman O., Maayan R., Weizman A., Weller A. (2006). Aggressive behavior and HPA axis hormones after social isolation in adult rats of two different genetic animal models for depression. Behav. Brain Res..

[7] Ko C.-Y., Liu Y.-P. (2016). Disruptions of sensorimotor gating, cytokines, glycemia, monoamines, and genes in both sexes of rats reared in social isolation can be ameliorated by oral chronic quetiapine administration. Brain Behav. Immun..

[8] Han X., Wang W., Shao F., Li N. (2011). Isolation rearing alters social behaviors and monoamine neurotransmission in the medial prefrontal cortex and nucleus accumbens of adult rats. Brain Res..

[9] Shao Y., Yan G., Xuan Y., Peng H., Huang Q.-J., Wu R., Xu H. (2015). Chronic social isolation decreases glutamate and glutamine levels and induces oxidative stress in the rat hippocampus. Behav. Brain Res..

[10] Murínová J., Hlaváčová N., Chmelová M., Riečanský I. (2017). The Evidence for Altered BDNF Expression in the Brain of Rats Reared or Housed in Social Isolation: A Systematic Review. Front. Behav. Neurosci..

[11] Toth M., Mikics E., Tulogdi A., Aliczki M., Haller J. (2011). Post-weaning social isolation induces abnormal forms of aggression in conjunction with increased glucocorticoid and autonomic stress responses. Horm. Behav..

[12] Amiri S., Haj-Mirzaian A., Rahimi-Balaei M., Razmi A., Kordjazy N., Shirzadian A., Mehr S.E., Sianati H., Dehpour A.R. (2015). Co-occurrence of anxiety and depressive-like behaviors following adolescent social isolation in male mice; possible role of nitrergic system. Physiol. Behav..

[13] Brenman J.E., Bredt D.S. (1997). Synaptic signaling by nitric oxide. Curr. Opin. Neurobiol..

[14] Nikonenko I., Boda B., Steen S., Knott G.W., Welker E., Muller D. (2008). PSD-95 promotes synaptogenesis and multiinnervated spine formation through nitric oxide signaling. J. Cell Biol..

[15] Nott A., Nitarska J., Veenvliet J.V., Schacke S., Derijck A.A.H.A., Sirko P., Muchardt C., Pasterkamp J., Smidt M., Riccio A. (2013). S-nitrosylation of HDAC2 regulates the expression of the chromatin-remodeling factor Brm during radial neuron migration. Proc. Natl. Acad. Sci. USA.

[16] Huang P.L., Dawson T.M., Bredt D.S., Snyder S.H., Fishman M.C. (1993). Targeted disruption of the neuronal nitric oxide synthase gene. Cell.

[17] Hardingham N., Dachtler J., Fox K. (2013). The role of nitric oxide in pre-synaptic plasticity and homeostasis. Front. Cell. Neurosci..

[18] Bernstein H.-G., Bogerts B., Keilhoff G. (2005). The many faces of nitric oxide in schizophrenia. A review. Schizophr. Res..

[19] Steinert J.R., Chernova T., Forsythe I. (2010). Nitric Oxide Signaling in Brain Function, Dysfunction, and Dementia. Neuroscientist.

[20] Joca S.R.L., Sartim A.G., Roncalho A.L., Diniz C.F., Wegener G. (2019). Nitric oxide signalling and antidepressant action revisited. Cell Tissue Res..

[21] Rodrigo J., Alonso D., Fernández A.P., Serrano J., Richart A., López J.C., Santacana M., Martínez-Murillo R., Bentura M.L., Ghiglione M. (2001). Neuronal and inducible nitric oxide synthase expression and protein nitration in rat cerebellum after oxygen and glucose deprivation. Brain Res..

[22] Sochocka M., Diniz B., Leszek J. (2017). Inflammatory Response in the CNS: Friend or Foe?. Mol. Neurobiol..

[23] Zhou Q.-G., Hua Y., Hu M., Luo C.-X., Han X., Zhu X.-J., Wang B., Xu J., Zhu D.-Y. (2007). Neuronal nitric oxide synthase contributes to chronic stress-induced depression by suppressing hippocampal neurogenesis. J. Neurochem..

[24] Lauer M., Johannes S., Fritzen S., Senitz D., Riederer P., Reif A. (2005). Morphological Abnormalities in Nitric-Oxide-Synthase-Positive Striatal Interneurons of Schizophrenic Patients. Neuropsychobiology.

[25] Nasyrova R.F., Ivashchenko D., Ivanov M.V., Neznanov N.G. (2015). Role of nitric oxide and related molecules in schizophrenia pathogenesis: Biochemical, genetic and clinical aspects. Front. Physiol..

[26] Bakshi V.P., Geyer M.A. (1999). Ontogeny of Isolation Rearing-Induced Deficits in Sensorimotor Gating in Rats. Physiol. Behav..

[27] Chmelova M., Balagova L., Marko M., Vrankova S., Cebova M., Jezova D., Riečanský I., Hlavacova N. (2019). Behavioral alterations induced by post-weaning isolation rearing of rats are accompanied by reduced VGF/BDNF/TrkB signaling in the hippocampus. Neurochem. Int..

[28] Klamer D., Engel J.A., Svensson L. (2004). The neuronal selective nitric oxide synthase inhibitor, Nω-propyl-l-arginine, blocks the effects of phencyclidine on prepulse inhibition and locomotor activity in mice. Eur. J. Pharmacol..

[29] Fejgin K., Pålsson E., Wass C., Svensson L., Klamer D. (2008). Nitric Oxide Signaling in the Medial Prefrontal Cortex is Involved in the Biochemical and Behavioral Effects of Phencyclidine. Neuropsychopharmacology.

[30] Issy A.C., Pedrazzi J.F.C., Yoneyama B.H., Del-Bel E.A. (2014). Critical role of nitric oxide in the modulation of prepulse inhibition in Swiss mice. Psychopharmacology.

[31] Ledo A., Frade J., Barbosa R.M., Laranjinha J. (2004). Nitric oxide in brain: Diffusion, targets and concentration dynamics in hippocampal subregions. Mol. Asp. Med..

[32] Moncada S., Bolanos J.P. (2006). Nitric oxide, cell bioenergetics and neurodegeneration. J. Neurochem..

[33] Issy A., Lazzarini M., Szawka R., Carolino R., Anselmo-Franci J., Del Bel E. (2011). Nitric oxide synthase inhibitors improve prepulse inhibition responses of Wistar rats. Behav. Brain Res..

[34] Swerdlow N.R., Geyer M.A. (1993). Clozapine and haloperidol in an animal model of sensorimotor gating deficits in schizophrenia. Pharmacol. Biochem. Behav..

[35] Braff D.L., Geyer M.A., Swerdlow N.R. (2001). Human studies of prepulse inhibition of startle: Normal subjects, patient groups, and pharmacological studies. Psychopharmacology.

[36] Khoja S., Asatryan L., Jakowec M.W., Davies D.L. (2019). Dopamine Receptor Blockade Attenuates Purinergic P2X4 Receptor-Mediated Prepulse Inhibition Deficits and Underlying Molecular Mechanisms. Front. Cell. Neurosci..

[37] Ralph R.J., Caine S.B. (2005). Dopamine D1 and D2 Agonist Effects on Prepulse Inhibition and Locomotion: Comparison of Sprague-Dawley Rats to Swiss-Webster, 129X1/SvJ, C57BL/6J, and DBA/2J Mice. J. Pharmacol. Exp. Ther..

[38] Komeima K., Hayashi Y., Naito Y., Watanabe Y. (2000). Inhibition of Neuronal Nitric-oxide Synthase by Calcium/Calmodulin-dependent Protein Kinase IIα through Ser847 Phosphorylation in NG108-15 Neuronal Cells. J. Biol. Chem..

[39] Tanda K., Nishi A., Matsuo N., Nakanishi K., Yamasaki N., Sugimoto T., Toyama K., Takao K., Miyakawa T. (2009). Abnormal social behavior, hyperactivity, impaired remote spatial memory, and increased D1-mediated dopaminergic signaling in neuronal nitric oxide synthase knockout mice. Mol. Brain.

[40] Zlatković J., Filipović D. (2013). Chronic social isolation induces NF-κB activation and upregulation of iNOS protein expression in rat prefrontal cortex. Neurochem. Int..

[41] Zlatković J., Bernardi R.E., Filipović D. (2014). Protective effect of Hsp70i against chronic social isolation stress in the rat hippocampus. J. Neural Transm..

[42] Madrigal J., Bueno B.G., Caso J., Perez-Nievas B.G., Leza J.C. (2006). Stress-Induced Oxidative Changes in Brain. CNS Neurol. Disord. Drug Targets.

[43] Zlatković J., Filipović D. (2012). Bax and B-cell-lymphoma 2 mediate proapoptotic signaling following chronic isolation stress in rat brain. Neuroscience.

[44] Filipović D., Zlatković J., Inta D., Bjelobaba I., Stojiljkovic M., Gass P. (2011). Chronic isolation stress predisposes the frontal cortex but not the hippocampus to the potentially detrimental release of cytochrome c from mitochondria and the activation of caspase-3. J. Neurosci. Res..

[45] Jiang B., Liang P., Deng G., Tu Z., Liu M., Xiao X. (2011). Increased stability of Bcl-2 in HSP70-mediated protection against apoptosis induced by oxidative stress. Cell Stress Chaperon.

[46] Didelot C., Schmitt E., Brunet M., Maingret L., Parcellier A., Garrido C. (2006). Heat Shock Proteins: Endogenous Modulators of Apoptotic Cell Death. Handb. Exp. Pharmacol..

[47] Kumar Y., Tatu U. (2003). Stress protein flux during recovery from simulated ischemia: Induced heat shock protein 70 confers cytoprotection by suppressing JNK activation and inhibiting apoptotic cell death. Proteomics.

[48] Kleppisch T., Feil R. (2009). cGMP Signalling in the Mammalian Brain: Role in Synaptic Plasticity and Behaviour. Handb. Exp. Pharmacol..

[49] Contestabile A. (2012). Role of Nitric Oxide in Cerebellar Development and Function: Focus on Granule Neurons. Cerebellum.

[50] Keinanen K., Wisden W., Sommer B., Werner P., Herb A., Verdoorn T., Sakmann B., Seeburg P. (1990). A family of AMPA-selective glutamate receptors. Science.

[51] Wang L., Zhu Z.-A. (2014). Nitric oxide show its survival role by NO-PKC pathway through cGMP-dependent or independent on the culture of cerebella granular neurons. Neurosci. Lett..

[52] Sammut S., Park D.J., West A.R. (2007). Frontal cortical afferents facilitate striatal nitric oxide transmission in vivo via a NMDA receptor and neuronal NOS-dependent mechanism. J. Neurochem..

[53] Rossetti Z.L., Crespi F. (2004). Inhibition of nitric oxide release in vivo by ethanol. Alcohol. Clin. Exp. Res..

[54] Hoque K.E., Indorkar R.P., Sammut S., West A.R. (2010). Impact of dopamine–glutamate interactions on striatal neuronal nitric oxide synthase activity. Psychopharmacology.

[55] Munhoz C., Bueno B.G., Madrigal J., Lepsch L., Scavone C., Leza J. (2008). Stress-induced neuroinflammation: Mechanisms and new pharmacological targets. Braz. J. Med. Biol. Res..

[56] Shal B., Khan A., Khan A., Ullah R., Ali G., Islam S., Haq I., Ali H., Seo E.-K., Khan S. (2021). Alleviation of Memory Deficit by Bergenin via the Regulation of Reelin and Nrf-2/NF-κB Pathway in Transgenic Mouse Model. Int. J. Mol. Sci..

[57] Perluigi M., Coccia R., Butterfield D.A. (2012). 4-Hydroxy-2-Nonenal, a Reactive Product of Lipid Peroxidation, and Neurodegenerative Diseases: A Toxic Combination Illuminated by Redox Proteomics Studies. Antioxid. Redox Signal..

[58] Khan A., Shal B., Khan A., Ullah R., Baig M., Haq I.U., Seo E., Khan S. (2021). Suppression of TRPV1/TRPM8/P2Y Nociceptors by Withametelin via Downregulating MAPK Signaling in Mouse Model of Vincristine-Induced Neuropathic Pain. Int. J. Mol. Sci..

[59] Herken H., Uz E., Özyurt H., Söğüt S., Virit O., Akyol O. (2001). Evidence that the activities of erythrocyte free radical scavenging enzymes and the products of lipid peroxidation are increased in different forms of schizophrenia. Mol. Psychiatry.

[60] Zhang X.Y., Tan Y.L., Cao L.Y., Wu G.Y., Xu Q., Shen Y., Zhou D.F. (2006). Antioxidant enzymes and lipid peroxidation in different forms of schizophrenia treated with typical and atypical antipsychotics. Schizophr. Res..

[61] Möller M., Du Preez J.L., Viljoen F.P., Berk M., Harvey B.H. (2013). N-acetyl cysteine reverses social isolation rearing induced changes in cortico-striatal monoamines in rats. Metab. Brain Dis..

[62] Meza C.A., La Favor J.D., Kim D.-H., Hickner R.C. (2019). Endothelial Dysfunction: Is There a Hyperglycemia-Induced Imbalance of NOX and NOS?. Int. J. Mol. Sci..

[63] Hlavacova N., Jezova D. (2008). Chronic treatment with the mineralocorticoid hormone aldosterone results in increased anxiety-like behavior. Horm. Behav..

[64] Geyer M.A., Swerdlow N.R. (2001). Measurement of Startle Response, Prepulse Inhibition, and Habituation. Curr. Protoc. Neurosci..

[65] Bredt D.S., Snyder S.H. (1990). Isolation of nitric oxide synthetase, a calmodulin-requiring enzyme. Proc. Natl. Acad. Sci. USA.

[66] Pecháňová O., Bernátová I., Pelouch V., Šimko F. (1997). Protein Remodelling of the Heart in NO-deficient Hypertension: The Effect of Captopril. J. Mol. Cell. Cardiol..

