# Supplementary material for: Duration of Social Isolation Affects Production of Nitric Oxide in the Rat Brain
Source: Int J Mol Sci. 2021 Sep 25;22(19):10340. doi: 10.3390/ijms221910340 (PMC8509065; doi:10.3390/ijms221910340)
Supplement: Supplementary file 1 [file ijms-22-10340-s001.zip › ijms-1381478-supplementary.pdf]

Duration of Social Isolation Affects Production of Nitric Oxide in the Rat Brain

Stanislava Vrankova [1,*], Zuzana Galandakova [1], Jakub Benko [1], Martina Cebova [1], Igor Riecansky [1,2,3] and Olga Pechanova [1]

[1] Centre of Experimental Medicine, Institute of Normal and Pathological Physiology,
Slovak Academy of Sciences, 841 04 Bratislava, Slovakia
[2] Social, Cognitive and Affective Neuroscience Unit, Department of Cognition, Emotion, and Methods in Psychology, Faculty of Psychology, University of Vienna, 1010 Vienna, Austria
[3] Department of Psychiatry, Slovak Medical University, 833 03 Bratislava, Slovakia

Supplementary tables

	Effect	*F*	*df*	*p*	η_p^2
	duration	2.384	(1, 34)	.132	0.066
**PPI 76db**	rearing	4.609	(1, 34)	**.039**	0.119
	duration * rearing	0.006	(1, 34)	.936	1.911
	duration	3.691	(1, 34)	.063	0.098
**startle reactivity**	rearing	1.801	(1, 34)	.189	0.050
	duration * rearing	0.910	(1, 34)	.347	0.026
	duration	0.276	(1, 34)	.603	0.008
**startle habituation**	rearing	0.230	(1, 34)	.635	0.007
	duration * rearing	2.414	(1, 34)	.129	0.066

Table S1. Prepulse inhibition (PPI) of the acoustic startle response at the prepulse intensity 76db, startle reactivity and startle habituation. Data were analysed using two-way ANOVA and subsequent post-hoc group comparisons using Sidak correction. Significant effects are marked in bold.

**NOS activity**	**Effect**	*F*	*df*	*p*	η_p^2
	duration	31.85	(1, 34)	**< .001**	0.484
cerebellum	rearing	0.21	(1, 34)	.650	0.006
	duration * rearing	18.83	(1, 34)	**< .001**	0.356
frontal cortex posterior to PFC	duration	101.54	(1, 34)	**< .001**	0.749
	rearing	0.23	(1, 34)	.633	0.006
	duration * rearing	18.18	(1, 34)	**< .001**	0.348

Table S2. NOS activity. Data were analysed using two-way ANOVA and subsequent post-hoc group comparisons using Sidak correction. Significant effects are marked in bold.

nNOS expression	Effect	F	df	p	η_p^2
cerebellum	duration	2.084	(1, 28)	.160	0.069
	rearing	0.291	(1, 28)	.594	0.010
	duration * rearing	0.749	(1, 28)	.394	0.026
frontal cortex	duration	0.185	(1, 28)	.671	0.007
	rearing	0.509	(1, 28)	.481	0.018
	duration * rearing	0.219	(1, 28)	.643	0.008
hippocampus	duration	24.051	(1, 28)	**< .001**	0.462
	rearing	0.645	(1, 28)	.429	0.023
	duration * rearing	2.125	(1, 28)	.156	0.071
striatum	duration	48.564	(1, 28)	**< .001**	0.634
	rearing	0.096	(1, 28)	.759	0.003
	duration * rearing	0.657	(1, 28)	.424	0.023

Table S3. nNOS expression. Data were analysed using two-way ANOVA and subsequent post-hoc group comparisons using Sidak correction. Significant effects are marked in bold.

iNOS expression	Effect	F	df	p	η_p^2
cerebellum	duration	5.754	(1, 28)	**.023**	0.170
	rearing	0.385	(1, 28)	.540	0.014
	duration * rearing	0.037	(1, 28)	.849	0.001
frontal cortex	duration	0.550	(1, 28)	.465	0.019
	rearing	0.047	(1, 28)	.831	0.002
	duration * rearing	0.332	(1, 28)	.569	0.012
hippocampus	duration	30.386	(1, 28)	**< .001**	0.520
	rearing	11.081	(1, 28)	**.002**	0.284
	duration * rearing	0.906	(1, 28)	.349	0.031
striatum	duration	8.898	(1, 28)	**.006**	0.229
	rearing	0.325	(1, 28)	.573	0.011
	duration * rearing	0.894	(1, 28)	.352	0.029

Table S4. iNOS expression. Data were analysed using two-way ANOVA and subsequent post-hoc group comparisons using Sidak correction. Significant effects are marked in bold.

**CD concentration**	**Effect**	***F***	***df***	***p***	$\boldsymbol{\eta_p^2}$
frontal cortex posterior to PFC	duration	145.88	(1, 34)	**< .001**	0.807
	rearing	0.09	(1, 34)	.772	0.002
	duration * rearing	6.07	(1, 34)	**.019**	0.148

Table S5. Concentration of conjugated dienes (CD). Data were analysed using two-way ANOVA and subsequent post-hoc group comparisons using Sidak correction. Significant effects are marked in bold.
